# Inhibition of Bladder Tumor Growth by Chitooligosaccharides in an Experimental Carcinogenesis Model

**DOI:** 10.3390/md10122661

**Published:** 2012-11-26

**Authors:** João C. Fernandes, José Sereno, Patricia Garrido, Belmiro Parada, Maria F. X. Cunha, Flávio Reis, Manuela E. Pintado, Alice Santos-Silva

**Affiliations:** 1 Institute for Molecular and Cellular Biology, Porto University, Porto 4150-180, Portugal; Email: assilva@ff.up.pt; 2 Institute of Pharmacology & Experimental Therapeutics, IBILI, Medicine Faculty, Coimbra University, Coimbra 3000-548, Portugal; Email: jose6sereno@hotmail.com (J.S.); apatricia.garrido@gmail.com (P.G.); freis@fmed.uc.pt (F.R.); 3 CBQF/Biotechnology School, Portuguese Catholic University, Porto 4200-072, Portugal; Email: mmpintado@esb.ucp.pt; 4 Department of Urology & Renal Transplantation, Coimbra University Hospital, Coimbra 3000-075, Portugal; Email: parada.belmiro@gmail.com; 5 Service of Anatomic Pathology, Coimbra University Hospital, Coimbra 3000-075, Portugal; Email: mfxcunha@gmail.com; 6 Department of Biochemistry, Pharmacy Faculty, Porto University, Porto 4050-313, Portugal

**Keywords:** chitosans, bladder cancer, chemoprevention, chitooligosaccharides

## Abstract

Urinary bladder cancer is one of the most common cancers worldwide, with the highest incidence in industrialized countries. Patients with cancer commonly use unconventional and complementary therapy including nutraceuticals. In this study we evaluated the efficacy of chitooligosaccharides (in orange juice) in rat bladder cancer chemoprevention and as therapeutic agent, on a rat model of urinary bladder carcinogenesis induced with *N*-butyl-*N*-(4-hydroxybutyl) nitrosamine. Results indicate that chitooligosaccharides may have a preventive effect on bladder cancer development and a curative effect upon established bladder tumors, dependent on the concentration ingested 500 mg/kg b.w., every three days, showed capacity to inhibit and prevent the proliferation of bladder cancer; however, this was associated with secondary effects such as hypercholesterolemia and hypertriglyceridemia. The use of lower doses (50 and 250 mg/kg b.w.) showed only therapeutic effects. It is further suggested that this antitumor effect might be due to its expected anti-inflammatory action, as well as by mechanisms not directly dependent of COX-2 inhibition, such as cellular proliferation control and improvement in antioxidant profile.

## 1. Introduction

**Figure 1:**
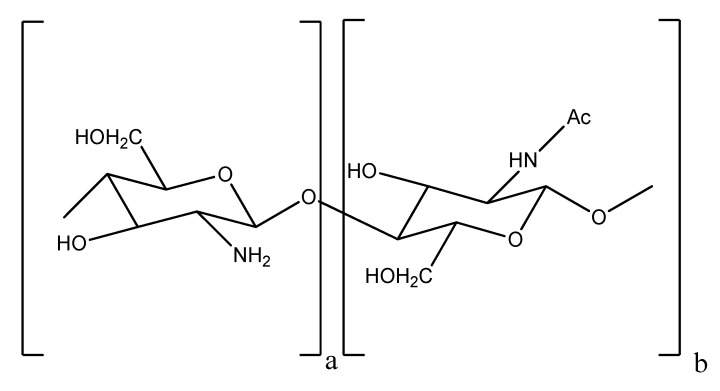
Structure of chitin and chitosan. Chitin is composed mainly by (b) units while chitosan is composed predominantly by (a) units (>50%). Ac—acetyl group.

Chitosan, a linear biopolymer comprising glucosamine and *N*-acetylglucosamine residues (Figure 1), is an *N*-deacetylated product of chitin, one of the most abundant polysaccharide in nature [1]. Due to its claimed properties such as antibacterial, antifungal, cholesterol lowering, antioxidant or antitumor properties, chitosan has been used as marine-derived nutraceutical in the food and food supplement industries [2,3]. However, its high molecular weight (MW)—which hampers solubility in acid-free aqueous media, has limited its practical applications [4]. Recent studies have focused on conversion of chitosan to oligosaccharides (termed chitooligosaccharides, COS)—because the latter are not only readily soluble in water due to their shorter chain lengths (generally, the MW of COS is 10 kDa or less) and free amino groups in D-glucosamine units, but also easily absorbed through the intestine, quickly getting into the blood flow [5,6,7]. The aforementioned properties, in addition to the positive charge of COS (which allows them to bind strongly to negatively charged surfaces), are responsible for many observed biological activities, such as lowering high blood pressure, controlling arthritis, treatment of diabetes mellitus and immuno-stimulation, in addition to prebiotic activity [6]. In addition, these oligosaccharides have also been reported as responsible for the inhibition of colorectal tumors, for example induced by dimethylhydrazine or azoxymethane, in the initiation and promotion stage [8,9,10]**.** Moreover, the development of sarcoma 180 tumor cells in mice was inhibited by oral treatment with COS [11]. Recently, chitosan oligosaccharides were shown to effectively inhibit uterine cervix carcinoma in BALB/c mice [6].

Urinary bladder (or bladder) cancer is one of the most common cancers worldwide, with the highest incidence in industrialized countries [12]. It is a huge concern for the medical community because of its malignancy, as well as high mortality and prevalence rates [12,13]. Furthermore, recurrence rates are also high, explaining that prevalence exceeds the primary incidence [14,15]. It is the fourth most common tumor in men and the eighth in women, accounting for 5%–10% of all malignancies in Western countries [12]. Excepting for superficial forms, bladder cancer prognosis is poor, particularly when lately diagnosed or inadequately treated, and is associated with high socioeconomic costs [13,16,17]. Despite the advances in medical care, the conventional methods of surgery, chemotherapy and radiotherapy have not impacted greatly on the general morbidity and mortality [18,19]. Thus, preventive strategies are crucial for the management and treatment of bladder cancer, which will depend on a better elucidation of the mechanisms underlying cancer lesion development. The carcinogen-induced model of bladder cancer with *N*-butyl-*N*-(4-hydroxybutyl) nitrosamine (BBN) in rats is a valuable experimental tool to study human cancer development, namely due to similar histological features with the human transitional cell carcinoma [20]. Urothelial transitional cell carcinoma is the predominant pathological lesion. The malignant transformation is a continuous process that includes dysplasic and proliferative epithelial abnormalities, pre-neoplasic changes and malignant lesions (papiloma and carcinoma) [12,21,22]. This model should also be important to evaluate the efficacy of modern therapeutic strategies, as it is the use of nutraceuticals [23]. An early treatment with agents that reverse these molecular and morphological pathways might hypothetically prevent the bladder cancer. Pre-clinical studies using green tea polyphenols, soy products, vitamins, selenium and nonsteroidal anti-inflammatory drugs demonstrate that bladder cancer is responsive to prevention strategies [24,25]. Others have also shown the efficacy of selective cyclooxygenase-2 inhibition for bladder cancer chemoprevention in rats [26].

Regarding the above features, in this research effort we intended to assess the anticarcinogenic effects of COS on a rat model of urinary bladder carcinogenesis induced with *N*-butyl-*N*-(4-hydroxybutyl) nitrosamine (BBN).

## 2. Results and Discussion

COS3 analysis indicated an average molecular weight (MW) of 1.763 ± 0.7 kDa, and a degree of deacetylation (DD) of 64.14 ± 1.96%.

**Figure 2:**
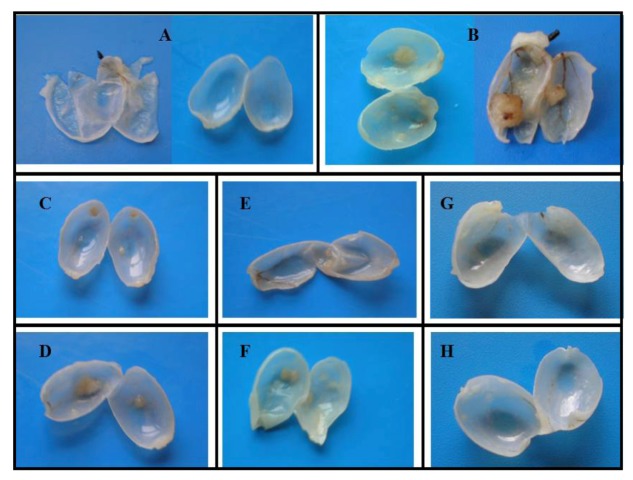
Macroscopic evaluation of the bladders, at the end of the 20 week-protocol: **A**—group control; **B**—group *N*-butyl-*N*-(4-hydroxybutyl) nitrosamine (BBN); **C**—group T-BBN + chitosan oligosaccharides (COS)(50); **D**—group P-COS(50) + BBN; **E**—group T-BBN + COS(250); **F**—group P-COS(250) + BBN; **G**—group T-BBN + COS(500); **H**—group P-COS(500) + BBN.

All 50 rats completed the 20 weeks protocol. No relevant changes were obtained between the groups during the study concerning body weight and beverage consumption, although at the end of the 20 weeks, rats fed with 500 mg/kg COS showed a slightly higher average weight (data not shown), but not statistically significant. Our results are in agreement with previous data from this model, concerning both the percentage of incidence of tumors and the type of lesions in the BBN rats [27], confirming the credibility and value of this model to evaluate chemopreventive efficacy of drugs. All formaldehyde pre-fixated bladders were opened and analyzed macroscopically for wall (urothelium) texture, thickness and vascularisation (Figure 2). 

The percentage of rats with tumor in each group, the number of tumors per rat with tumor, as well as the mean tumor volume per rat and per tumor were then evaluated macroscopically (Table 1). All the bladders from the group control have revealed a pattern of normality, with absence of any type of malignity. The wall texture, thickness and vascularisation were normal (Figure 2A). Similar profile was found for the COS control groups (groups COS(250) and COS(500)), with limpid, translucent and tiny bladders, without presence of any abnormal mass or vascularisation (data not shown). In the group BBN, however, 80% of the rats had bladder tumors, all easily observed due to their large dimensions. The bladder walls were thicker, with new or enlarged small vessels, suggesting neo-angiogenesis, and there was unequivocal formation of tumor, some of them of relevant volume (Figure 2B). In groups P-COS(50) + BBN and P-COS(250) + BBN, all bladders showed a similar pattern to those seen for the BBN group—bladder walls were thicker, new small vessels suggested neo-angiogenesis and unequivocal formation of tumors, when compared to control group (Figure 2A), signifying that at these concentrations (*i.e.*, 50 and 250 mg/kg) COS have insignificant effects preventing bladder cancer (Figure 2C,F). 

In groups T-BBN + COS(50) and T-BBN+COS(250) we observed a dose-dependent therapeutic effect—a higher treatment dose resulted in higher treatment effectiveness; the mean number of tumors per rat and the mean number of tumors per rat with tumor, were significantly lower on both groups, compared to group BBN; the volume per tumor was also considerably lower (Figure 2D,E); however, all the described parameters were significantly lower in group T-BBN + COS(250), compared to group T-BBN + COS(50).

**Table 1:** Results of the macroscopic (quantitative) and microscopic (qualitative) evaluation of urothelium lesions.

Rat Groups (*n* = 5 each)	1	2	3	4	5	6	7	8	9	10
**Macroscopic analysis**										
Number of tumors										
% of tumors/group	0	40	100	0	20	80	0	20	25	80
Total no. of tumors	0	2	9	0	1	5	0	1	2	9
No. of tumors/rat	0	0.4	1.8	0	0.2	1	0	0.2	0.4	1.8
Tumor volume										
Per tumor (mm^3^)	0	2.6	3.22	0	1.04	3.25	0	1.04	1.3	4,49
**Microscopic (histological) analysis**										
Pre-neoplastic lesions										
Hyperplasia	0	1/2	6/9	0	0	2/5	0	0	0	5/9
High-grade dysplasia	0	1/2	3/9	0	1/1	2/5	0	1/1	0	4/9
Malignant lesions, tumor										
Papillary	0	1/2	9/9	0	1/1	4/4	0	1/1	1/2	7/9
Infiltrative	0	0	0	0	0	0	0	0	0	2/9
Tumor grading										
Low grade (G1)	0	1/2	5/9	0	0	3/4	0	0	1/2	3/9
High grade (G2/G3)	0	1/2	4/9	0	1/1	1/4	0	1/1	1/2	6/9

Values are mean (SEM) or *n* variable. **1**: control; **2**: T-BBN + COS(50); **3**: P-COS(50) + BBN; **4**: COS(250); **5**: T-BBN + COS(250); **6**: P-COS(250) + BBN; **7**: COS(500); **8**: T-BBN + COS(500); **9**: P-COS(500) + BBN; and **10**: BBN.

In both groups T-BBN + COS(500) and P-COS(500) + BBN, a marked inhibition on bladder tumor growth was observed (Figure 2G,H). Although the preventive group (group P-COS(500) + BBN) showed higher efficiency, both groups reduced significantly the number of tumors and respective volume, when compared to BBN group; in fact, this was the only concentration where a preventive effect was seen.

**Figure 3:**
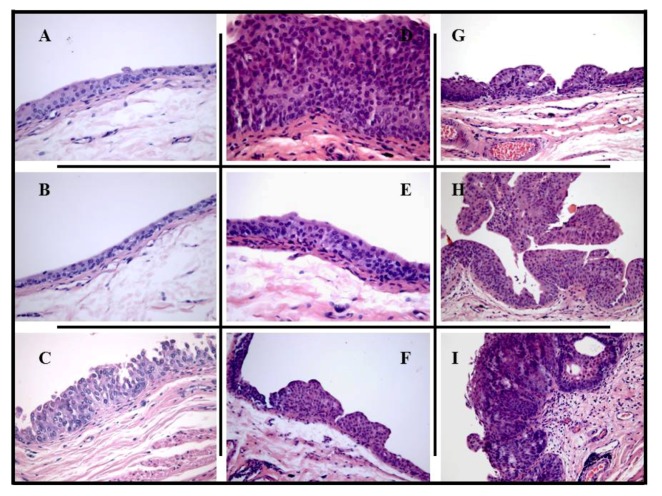
Microscopic histomorphology (H & E). The typical bladder from control group (**A**) and control groups with COS (**B**) treated-rats had no signs of pre-neoplasic lesions or gross tumor formation. In the carcinogen groups, the bladder from several animals presented hyperplasia (**C** and **D**) (groups T-BBN + COS(50), P-COS(250) + BBN and BBN) and high-grade dysplasia (**E** and **F**) (groups T-BBN + COS(50), P-COS(50) + BBN, T-BBN + COS(250), P-COS(250) + BBN, T-BBN + COS(500) and BBN), including those without tumor formation, as well as malignant lesions, such as papillary tumors (**G** and **H**) (groups 2, 3, 5, 6, 8, 9 and 10) or infiltrative (**I**) (group 10).

Histological examination (Figure 3) showed less aggressive histological changes in all groups treated with COS than in the control group with BBN (group BBN). The bladder from negative control animals (group control) had no signs of pre-neoplasic lesions (neither hyperplasia nor dysplasia), as well as those from the COS control groups (groups COS(250) and COS(500)). In the carcinogen (BBN) group, there was evident malignant transformation, including hyperplasia and dysplasia, present in all the animals, including the one without tumor formation. Furthermore, there were also malignant lesions—papillary and infiltrative (Table 1).

The use of COS as therapeutic has notoriously decreased bladder cancer progression, at all tested concentrations (*i.e.*, groups T-BBN + COS(50), T-BBN + COS(250) and T-BBN + COS(500)). In these groups, whose urothelia only demonstrated hyperplasia (only one animal in group T-BBN + COS(50)) and dysplasia (one animal in each of the three groups), the few tumors seen were all papillary. When used in a preventive way, no pre-neoplastic lesions were observed at 500 mg/kg (group P-COS(500) + BBN), with only one papillary tumor. However, at lower doses (group P-COS(50) + BBN and P-COS(250) + BBN) the number of malignant lesions was much higher, nevertheless all tumors were papillary. Histological examination of these two groups showed, opposite to macroscopic examination, some preventive effect, but almost negligible compared to group P-COS(500) + BBN.

The antiproliferative activity of COS was reported in several preclinical studies, including cell-line cultures of human leukemia and breast cells and *in vivo* studies upon mouse sarcoma-180, fibrosarcoma, uterine cervix tumor or Ehrlich ascites tumors in rats and mice [6,28,29,30,31]. The mechanisms underlying the anticarcinogenic properties of COS remain to be fully elucidated, but the most accepted mechanism states that COS may inhibit the growth of tumor cells by immunoenhancement rather than directly damaging the tumor cells. It is suggested that the antitumor activity of water soluble chitosans and COS might be related, in part, to an enhancement of the proliferation of cytolytic lymphocytes—natural killer cells [6,11]. However, lymphocytes count in this study did not reveal any significant difference, and therefore does not sustain this hypothesis (data not shown). 

It is largely accepted that the mechanisms underlying cancer appearance and progression are multifactorial. Inflammation, through its mediators (cytokines and other growth factors), seems to be one of the main contributors for cancer growth. One of the mechanisms more closely linked to inflammation is the pathway of COX enzymes, in particular COX-2 [32], whose levels and activity have been reported to be elevated in several types of cancers [33]. A chemopreventive role for COX-2 inhibition in bladder cancer was previously reported in animal models [34,35,36], but the mechanisms by which these compounds are able to act on carcinogenesis remain to be elucidated. Furthermore, a key role for COX-2 in carcinogenesis has been given by the positive effect of its down-regulation on tumors incidence both in clinical and experimental studies for distinct types of tumors, including the bladder cancer [37,38,39,40]. In a previous study we concluded that COS possess anti-inflammatory activity involving the inhibition of the cyclooxygenase pathway [41]. In addition, Lee *et al.*, also reported that COS may exert an anti-inflammatory effect via down-regulation of transcriptional and translational expression levels of COX-2 [42]. Therefore, it is suggested here that the anticarcinogenic activity shown by COS may be related to its capacity to down-regulate expression levels of COX-2. This hypothesis merits further elucidation, which could be done assessing COX-2 expression and activity.

The lipid profile showed that a 500 mg/kg COS dose caused hypercholesterolaemia and hypertriglyceridaemia in all the three groups treated with this dose (COS(500), T-BBN + COS(500) and P-COS(500) + BBN) (Table 2). In addition, liver weight was considerably heavier than in the control group (*P* < 0.01) (data not shown). Rats in group P-COS(250) + BBN presented changes in hepatic function, with significantly elevated values of AST and ALT. No other significant biochemical or organ weight changes were perceived. Concerning hematology parameters (data not shown), groups COS(500), T-BBN + COS(500) and P-COS(500) + BBN presented higher values for hematocrit, red blood cells count and hemoglobin, however, were statistically not significant. In the opposite position, group P-COS(250) + BBN showed lower values for the same parameters than the other groups, though only the difference on reticulocyte values was significant. 

**Figure 4:**
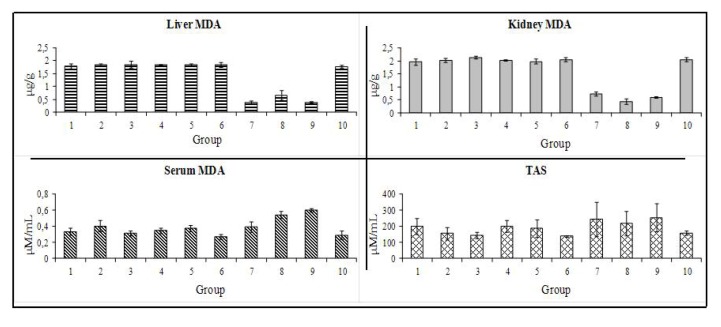
Redox status markers: lipidic peroxidation (MDA content) in liver (**A**), kidney (**B**) and serum (**C**); serum TAS (**D**).

The liver and kidney malondialdehyde (MDA) content, a lipidic peroxidation marker, was unchanged between the control and the BBN groups (Figure 4). In addition, groups treated with 50 and 250 mg/kg of COS also showed similar MDA profile, however, in the groups given a 500 mg/kg dose (*i.e.*, groups COS(500), T-BBN + COS(500) and P-COS(500) + BBN), there was a significant decrease in both liver (*p* < 0.001) and kidney (*p* < 0.001) MDA content. Serum MDA concentration showed an opposite trend, since in this case the 500 mg/kg groups (groups T-BBN + COS(500) and P-COS(500) + BBN) showed significantly higher values (*p* < 0.05) than both control groups. Total antioxidant status (TAS) did not present significant differences between the 10 groups. However, the positive control group (*i.e.*, group BBN) and groups T-BBN + COS(50), P-COS(50) + BBN and P-COS(250) + BBN showed lower TAS values when compared with all the other groups.

**Table 2:** Biochemical data for the 10 groups at the end of the study (week 20).

	Group										
Markers		1	2	3	4	5	6	7	8	9	10
Urea ^#^		15.45	14.62	17.22	19.82	16 (±1.21)	16.47	15.5	15.63	15.73	15.55
		(±0.95)	(±0.82)	(±1.39)	(±1.26)		(±0.37)	(±0.99)	(±1.59)	(±0.67)	(±0.76)
Creat *		0.42	0.39	0.38	0.5	0.46	0.4	0.40	0.39	0.40	0.45
		(0.46–0.38)	(0.46–0.32)	(0.41–0.35)	(0.52–0.49)	(0.52–0.41)	(0.43–0.39)	(0.43–0.34)	(0.42–0.33)	(0.44–0.37)	(0.47–0.43)
Uric Acid *		0.4	0.35	0.6	1	1	0.5	0.58	0.5	0.58	0.5
		(0.52–0.3)	(0.47–0.27)	(0.7–0.5)	(1.05–0.9)	(1.35–0.75)	(0.55–0.45)	(0.63–0.5)	(0.55–0.45)	(0.63–0.55)	(0.55–0.48)
G.P.T. *		42	37.5	47	51	45	132.5	30.75	24.25	29	49.5
		(43.5–40.5)	(39.3–36.8)	(49.5–46)	(53.8–49.5)	(46–42.5)	(141.8–115)	(36.25–25)	(25.5–21.8)	(29.8–28.3)	(54–45.25)
G.O.T. *		64	66	67	75.5	67	213	69	72	69.75	82
		(68–60.5)	(72.8–63.8)	(69–65)	(82.5–73.5)	(69.5–64.5)	(237.8–193)	(71.8–66.8)	(80.8–64.8)	(75–60.75)	(84.3–79.3)
α-Amylase ^#^		547.5	500.75	510.5	544.5	453.75	459.75	531.75	552	551.5	545
		(±106.96)	(±79.78)	(±25.44)	(±23.9)	(±34.97)	(±39.79)	(±15.84)	(±31.51)	(±115.23)	(±77.18)
Cholesterol *		47.5	42.5	45	46	43.5	39.5	62.25	57.25	63	42
		(50–42.25)	(43.5–41)	(46–44)	(47.3–44.3)	(47–39.5)	(41.5–38.3)	(69.5–55.3)	(63.25–51)	(69–57.5)	(43.3–40.5)
HDL *		27.5	22	27	28	24	23	36.75	33	35.5	24
		(29–25.5)	(23.3–21.8)	(27.25–27)	(28.8–27.3)	(25.3–22)	(24–23)	(40.3–33)	(35.5–31)	(38.8–31.8)	(24.5–23.5)
LDL *		13	14.5	12	11.5	13.5	12.5	1.68	1.73	1.8	13.5
		(15–11.75)	(16–12.75)	(13.5–11)	(12–11)	(15–12.75)	(13–12)	(1.7–1.68)	(1.8–1.68)	(1.83–1.78)	(14.5–12.8)
Atherogenic *		1.8	1.85	1.65	1.65	1.9	1.7	20.75	19	17.5	1.75
		(1.9–1.7)	(1.93–1.78)	(1.7–1.6)	(1.7–1.6)	(1.9–1.875)	(1.7–1.67)	(24–18.25)	(20–17.5)	(18.8–15.8)	(1.8–1.7)
Trigly *		75	74.5	48	59.5	55.5	34.5	134	122.5	128.75	50
		(86–67)	(78.75–67)	(52.3–44.5)	(65.7–51.8)	(59.8–49.7)	(41–28.25)	(136–130.8)	(123.5–115)	(134.3–126)	(53.5–40.8)

* Non-Gaussian distribution (median–inter-quartile range); ^#^ Normal distribution (mean–standard deviation). **1**: negative control group; **2**: 50 mg/kg COS therapeutic group; **3**: 50 mg/kg COS preventive group; **4**: 250 mg/kg COS control group; **5**: 250 mg/kg COS therapeutic group.; **6**: 250 mg/kg COS preventive group; **7**: 500 mg/kg COS control group; **8**: 500 mg/kg COS therapeutic group; **9**: 500 mg/kg COS preventive group; and **10**: BBN control group. G.P.T.—Glutamic-Pyruvic Transaminase; G.O.T.—Glutamic-Oxaloacetic Transaminase.

In this study, apart from the above reported anti-proliferative effect, the positive impact of COS3 on bladder cancer chemoprevention/treatment seems also to be related with a beneficial influence on redox status. Even though the lower values of MDA in serum, the positive effect of a 500 mg/kg treatment seem to be extended to the redox status, given by the remarkable shift to the antioxidant capacity in the liver and kidney, as well as a high capacity avoid reactive oxygen species (ROS) formation.

Inflammation, proliferation and oxidative stress seem to be important for bladder carcinogenesis [30]. We have demonstrated in this study that the use of COS3, in the stage of cancer initiation, or even before, as chemopreventive therapy, has a notable effect dose dependent, on cancer appearance and progression, which might be particularly useful for individuals of higher risk as well as for patients treated for an previous episode of bladder cancer and under high risk of recurrence [14,15].

## 3. Experimental Section

### 3.1. COS Characterization (MW and DD)

COS derived from crab shells, named COS3, were purchased from Nicechem (Shanghai, China). The average MW of COS3 was assessed by size exclusion chromatography (SEC). Two combined TSKGel series columns (G2500PW_XL_ × G5000PW_XL_) together with a PW_XL_ guard column were used, coupled with a RID-10A Shimadzu refractive index (RI) detector. A flow rate of 0.8 mL·min^−1^ and a mobile phase solution of 0.5 M AcOH–0.2 M AcONa at pH 4.4–4.5 (25 °C), were found to be the most suitable conditions to evaluate COS molecular weight. Pullulan (TOSOH Biosciences) of different molecular weights were used as standards to calibrate the column, and quantification of COS was performed by external calibration, using chitobiose as standard. Data provided by the SEC-HPLC system were collected and analyzed using the Chromeleon system version 6.7. The DD was determined using a FT-IR Perkin Elmer infrared spectrometer. An aliquot of COS sample was mixed with potassium bromide (1:1000) and compressed into pellets. The IR spectra were recorded and the absorbance values of the suitable absorption bands were calculated using the base line method. The DD was calculated from the value of the absorption band ratio A_amide peak_/A_reference peak_. A number of absorption band ratios have been proposed in the literature, differing either in the band selected as in the internal reference band. One such band ratio is A_1655_/A_3450_, determined using a line draw from 4000 cm^−1^ to 2500 cm^−1^ as the base line for the hydroxyl group band, and one drawn from 1800 cm^−1^ to 1600 cm^−1^ as the base line for the amide I band. The DD was thus calculated according to the following equation [43],

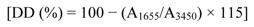
(1)


### 3.2. Animals and Treatment

Fifty male Wistar rats (Charles River Laboratory Inc, Barcelona, Spain), 250–285 g, 8 weeks old, were maintained in an air-conditioned room, subjected to 12-h dark/light cycles and given standard laboratory rat chow (IPM-R20, Letica, Barcelona, Spain) and free access to tap water. Animal experiments were conducted according to the European Communities Council Directives on Animal Care. The rats were divided into 10 groups (*n* = 5 each): **1**, Control group—orange juice only; **2**, T-BBN + COS(50)—treatment group receiving daily 0.05% BBN and 50 mg/kg of COS in every 3 days; **3**, P-COS(50) + BBN—preventive group receiving 50 mg/kg in every 3 days of COS and 0.05% BBN; **4**, COS(250) Control—receiving only 250 mg/kg of COS in every 3 days; **5**, T-BBN + COS(250)—treatment group receiving daily 0.05% BBN and 250 mg/kg of COS in every 3 days; **6**, P-COS(250) + BBN—preventive group receiving 250 mg/kg in every 3 days of COS and 0.05% BBN; **7**, COS(500) Control—receiving only 500 mg/kg of COS in every 3 days; **8**, T-BBN + COS(500)—treatment group receiving daily 0.05% BBN and 500 mg/kg of COS in every 3 days; **9**, P-COS(500) + BBN—preventive group receiving 500 mg/kg in every 3 days of COS and 0.05% BBN; and **10**, BBN group—carcinogen group receiving 0.05% BBN (Tokyo Chemical Industry Co., Ltd., Tokyo, Japan). The experimental study was conducted in two steps, a tumor-induction phase from week 1 to week 8, when rats from groups 2, 3, 5, 6, 8, 9 and 10 received BBN *ad libitum *in orange juice (preventive groups—3, 6 and 9, received COS at the same time), and a treatment phase from week 8 to week 20, when rats in groups 2, 5 and 8 received COS at different concentrations by an oesophageal cannula. All rats completed the 20-week study protocol, and body weight and amount of drunken liquid were monitored during the experimental period. All procedures involving animals were in accordance with the Association for Pharmacology and Experimental Therapeutics, and approved by the Institutional Ethics Committee of the Faculty of Medicine from the University of Coimbra. Approval ID: FMUC/09/10.

### 3.3. Blood and Organs Collection

At the end of treatment the rats were anaesthetised intraperitoneal with 2.0 mg/kg of a 2:1 (v:v) 50.0 mg/mL ketamine (Ketalar^®^, Parke-Davis, Pfizer Laboratories Lda, Seixal, Portugal) solution in 2.5% chlorpromazine (Largatil^®^, Rhône-Poulenc Rorer, Vitória Laboratories, Amadora, Portugal). Blood samples were immediately collected by venepuncture from the jugular vein in needles with no anticoagulant (for serum samples collection) or with EDTA. Under anaesthesia the rats were killed by cervical dislocation, and the liver, kidneys and heart immediately removed, weighed and placed in ice-cold Krebs buffer or formaldehyde for further analysis. Before removal, bladders were intraluminally injected with a buffered formaldehyde solution as pre-fixation for histological analyses. A ligature was placed around the bladder neck to maintain proper distension. 

### 3.4. Blood Analysis

Several haematological variables were measured in EDTA-whole blood using an automatic Coulter Counter^®^ (Beckman Coulter Inc., Foster City, CA, USA), *i.e.*, a red blood cell count, hematocrit, haemoglobin concentration, platelet and white blood cells count.

### 3.5. Macroscopic Tumor Analysis

For a macroscopic quantitative analysis (number and volume of tumors), each bladder pre-fixed in formaldehyde was carefully opened, the lumen inspected for grossly visible lesions and the number of tumors per rat and the volume of each tumor were recorded to calculate the incidence of tumor per group and the mean volume per rat. A tumor was defined as a lesion of >0.5 mm in diameter. 

### 3.6. Microscopic Tumor Analysis

For the microscopic qualitative analysis (bladder histology), the bladder was immersion-fixed in 4% buffered formaldehyde and processed for paraffin sectioning [44]. Three slices from each bladder were embedded, 3 μm sections cut and stained with hematoxylin and eosin (H & E), and examined histologically by one author (Cunha MFX) unaware of the treatments. 

### 3.7. Antioxidant Capacity

Serum redox status was assessed by two methods: a thiobarbituric acid reactive species assay, in which serum was used to determine the products of lipid peroxidation, *i.e.*, malondialdehyde (MDA), as previously described [45]; and a ferric reducing antioxidant potential (FRAP) assay, in which serum antioxidant capacity was measured as FRAP, as previously described [46], and termed the total antioxidant status (TAS).

### 3.8. Statistical Analysis

Mean values and standard deviations were accordingly calculated from the obtained experimental data. Because several of the studied variables presented a non-Gaussian distribution, we present that data as median (interquartile range) values. For statistical analysis, we used the Statistical Package for Social Sciences (SPSS) version 15.0 for Windows. To evaluate the differences between groups, we used the nonparametric Kruskal-Wallis *H *test. When statistical significance was achieved, single comparisons (two groups) were made by the use of the Mann-Whitney *U *test. A *p *value of <0.05 was considered to be statistically significant.

## 4. Conclusions

In conclusion, our study demonstrates that application of COS3 has a preventive effect on bladder cancer appearance, as well as it can be successfully used as a curative beneficial ingredient, dependent on the concentration. This antitumor effect might be due to its expected anti-inflammatory action, in addition to other mechanisms not directly dependent of COX-2 inhibition, such as cellular proliferation control and improvement in antioxidant profile. 

Although this was a preliminary study, the results are promising and justify further investigation, including other dosages, to validate the role of cancer chemoprevention strategies based on the therapeutic use of COS, as well as to further investigate possible secondary effects, mainly in liver.

## References

[1] Lin C.W., Chen L.J., Lee P.L., Lee C.I., Lin C.J., Chiu J.J. (2007). The inhibition of TNF-α-induced E-selectin expression in endothelial cells via the JNK/NF-κB pathways by highly *N*-acetylated chitooligosaccharides. Biomaterials.

[2] Vernazza C.L., Gibson G.R., Rastall R.A. (2005). *In vitro* fermentation of chitosan derivatives by mixed cultures of human faecal bacteria. Carbohydr. Polym..

[3] Rasmussen R.S., Morrissey M.T., Shahidi F., Barrow C. (2008). Marine Nutraceuticals and Functional Foods.

[4] Roller S., Covill N. (1999). The antifungal properties of chitosan in laboratory media and apple juice. Int. J. Food Microbiol..

[5] Vårum K.M., Ottøy M.H., Smidsrød O. (1994). Water-solubility of partially *N*-acetylated chotosans as a function of pH, effect of chemical composition and depolymerisation. Carbohydr. Polym..

[6] Kim S.K., Rajapakse N. (2005). Enzymatic production and biological activities of chitosan oligosaccharides (COS): A review. Carbohydr. Polym..

[7] Chae S.Y., Jang M.K., Nah J.W. (2005). Influence of molecular weight on oral absorption of water soluble chitosans. J. Control Release.

[8] Reddy B.S., Hamid R., Rao C.V. (1997). Effect of dietary oligofructose and inulin on colonic preneoplastic aberrant crypt foci inhibition. Carcinogenesis.

[9] Wijnands M.V.W., Appel M.J., Hollanders V.M.H., Woutersen R.A.A. (1999). Comparison of the effects of dietary cellulose and fermentable galacto-oligosaccharide, in a rat model of colorectal carcinogenesis, fermentable fibre confers greater protection than non-fermentable fibre in both high and low fat backgrounds. Carcinogenesis.

[10] Wijnands M.V.W., Schoterman H.C., Bruijntjes J.P., Hollanders V.M.H., Wouterse R.A. (2001). Effect of dietary galacto-oligosaccharides on azoxymethane-induced aberrant crypt foci and colorectal cancer in Fischer 344 rats. Carcinogenesis.

[11] Maeda Y., Kimura Y. (2004). Antitumor effects of various low molecular-weight chitosans are due to increased natural killer activity of intestinal intraepithelial lymphocytes in sarcoma 180-bearing mice. Nutr. Cancer.

[12] Grasso M. (2008). Bladder cancer: A major public health issue. Eur. Urol. Suppl..

[13] Ferlay J., Autier P., Boniol M., Heanue M., Colombet M., Boyle P. (2007). Estimates of the cancer incidence and mortality in Europe in 2006. Ann. Oncol..

[14] Pisani P., Bray F., Parkin D.M. (2002). Estimates of world-wide prevalence of cancer for 25 sites in the adult prevalence. Int. J. Cancer.

[15] Sylvester R.J., van der Meijden A.P., Oosterlinck W., Witjes J.A., Bouffioux C., Denis L., Newling D.W., Kurth K. (2006). Predicting recurrence and progression in individual patients with stage Ta T1 bladder cancer using EORTC risk tables, a combined analysis of 2596 patients from seven EORTC trials. Eur. Urol..

[16] Kirkali Z., Chan T., Manoharan M., Algaba F., Busch C., Cheng L., Kiemeney L., Kriegmair M., Montironi R., Murphy W.M. (2005). Bladder cancer: Epidemiology, staging, grading and diagnosis. Urology.

[17] Sangar V.K., Ragavan N., Matanheleia S.S., Watson M.W., Blades R.A. (2005). The economic consequences of prostate and bladder cancer in UK. BJU Int..

[18] Malkowicz S.B., van Poppel H., Mickisch G., Pansadoro V., Thüroff J., Soloway M.S., Chang S., Benson M., Fukui I. (2007). Muscle-invasive urothelial carcinoma of the bladder. Urology.

[19] Evans C., Debruyne F., Payne H., Solsona E., Teillac P., Tubaro A. (2007). Bladder cancer, management and future directions. Eur. Urol. Suppl..

[20] Fukushima S., Hirose M., Tsuda H., Shirai T., Hirao K. (1976). Histological classification of urinary bladder cancers in rats induced by *N*-butyl-*N*-(4-hydroxybutyl) nitrosamine. Gann.

[21] Montironi R., Mazzucchelli R. (1976). Preneoplastic Lesions and Conditions of the urinary bladder. EAU Update Ser..

[22] Sauter G., Algaba F., Amin M., Busch C., Cheville J., Gasser T., Grignon D.J., Hofstadter F., Lopez-Beltran A., Epstein J.I., Eble J.N., Sauter G., Epstein J.L., Sesterhenn I. (2004). WHO Classification of Tumors of the Urinary System and Male Genital Organs.

[23] Gofrit O.N., Birman T., Ayesh S., Ohana P., Hochberg A. (2006). Chemically induced bladder cancer—A sonographic and norphologic description. Urology.

[24] Hameed D.A., el-Metwally T.H. (2008). The effectiveness of retinoic acid treatment in bladder cancer, impact on recurrence, survival and TGF-alpha and VEGF as end-point biomarkers. Cancer Biol. Ther..

[25] Leppert J.T., Shvarts O., Kawaoka K., Lieberman R., Belldegrun A.S., Pantuck A.J. (2006). Prevention of bladder cancer: A review. Eur. Urol..

[26] Parada B., Sereno J., Reis F., Teixeira-Lemos E., Garrido P., Pinto A.F., Cunha M.F., Pinto R., Mota A., Figueiredo A., Teixeira F. (2009). Anti-inflammatory, anti-proliferative and antioxidant profiles of selective cyclooxygenase-2 inhibition as chemoprevention for rat bladder carcinogenesis. Cancer Biol. Ther..

[27] Hattori K., Iida K., Joraku A., Tsukamoto S., Akaza H., Oyasu R. (2006). Chemopreventive effects of cyclooxygenase-2 inhibitor and epidermal growth factor-receptor kinase inhibitor on rat urinary bladder carcinogenesis. BJU Int..

[28] Nam K.S., Shon Y.H. (2009). Suppression of metastasis of human breast cancer cells by chitosan oligosaccharides. J. Microbiol. Biotechnol..

[29] Suzuki K., Mikami T., Okawa Y., Tokoro A., Suzuki S., Suzuki M. (1986). Antitumor effect of hexa-*N*-acetylchitohexaose and chitohexaose. Carbohydr. Res..

[30] Tokoro A., Tatewaki N., Suzuki K., Mikami T., Suzuki S., Suzuki M. (1988). Growth-inhibitory effect of hexa-*N*-acetylchitohexaose and chitohexaose against Meth-A solid tumor. Chem. Pharm. Bull..

[31] Wang J., Chen Y., Ding Y., Shi G., Wan C. (2008). Research of the degradation products of chitosan’s angiogenic function. Appl. Surf. Sci..

[32] Kam P.C., See A.U. (2000). Cyclo-oxygenase isoenzymes, physiological and pharmacological role. Anaesthesia.

[33] Harris R.E. (2007). Cyclooxygenase-2 (COX-2) and the inflammogenesis of cancer. Subcell. Biochem..

[34] DeGortari A.E., Han C., Glickman L.T. (2000). Cisplatin *versus *cisplatin combined with piroxicam in a canine model of human invasive urinary bladder cancer. Cancer Chemother. Pharmacol..

[35] Grubbs C.J., Lubet R.A., Koki A.T., Leahy K.M., Masferrer J.L., Steele V.E., Kelloff G.J., Hill D.L., Seibert K. (2000). Celecoxib inhibits *N*-Butyl-*N*-(4-hydroxybutyl) nitrosamine induced urinary bladder cancers in Male B6D2F1 mice and female Fischer-344 rats. Cancer Res..

[36] Okajima E., Denda A., Ozono S., Takahama M., Akai H., Sasaki Y., Kitayama W., Wakabayashi K., Konishi Y. (1998). Chemopreventive effects of nimesulide, a selective cyclooxygenase-2 inhibitor, on the development of rat urinary bladder carcinomas initiated by *N*-butyl-*N*-(4-hydroxybutyl) nitrosamine. Cancer Res..

[37] De Heer P., Sandel M.H., Guertens G., de Boeck G., Koudijs M.M., Nagelkerke J.F., Junggeburt J.M., de Bruijn E.A., van de Velde C.J., Kuppen P.J. (2008). Celecoxib inhibits growth of tumors in a syngeneic rat liver metastases model for colorectal cancer. Cancer Chemother. Pharmacol..

[38] Keller J.J., Giardiello F.M. (2003). Chemoprevention strategies using NSAIDs and COX-2 inhibitors. Cancer Biol. Ther..

[39] Narayanan N.K., Nargi D., Horton L., Reddy B.S., Bosland M.C., Narayanan B.A. (2009). Inflammatory processes of prostate tissue microenvironment drive rat prostate carcinogenesis, preventive effects of celecoxib. Prostate.

[40] Ristimäki A., Nieminen O., Saukkonen K., Hotakainen K., Nordiling S., Haglund C. (2001). Expression of cyclooxygenase-2 in human transitional cell carcinoma of the urinary bladder. Am. J. Pathol..

[41] Fernandes J.C., Spindola H., de Sousa V., Santos-Silva A., Pintado M.E., Malcata F.X., Carvalho J.E. (2010). Anti-inflammatory activity of chitooligosaccharides *in vivo*. Mar. Drugs.

[42] Lee S.H., Senevirathne M., Ahn C.B., Kim S.K., Je J.Y. (2009). Factors affecting anti-inflammatory effect of chitooligosaccharides in lipopolysaccharides-induced RAW264.7 macrophage cells. Bioorg. Med. Chem. Lett..

[43] Baxter A., Dillon M., Taylor K.D.A., Robert G.A.F. (1992). Improved method for i.r. determination of the degree of *N*-acetylation of chitosan. Int. J. Biol. Macromol..

[44] El-kott A.F. (2007). Flow cytometry and KI67 expression in rat’s urinary bladder carcinogenesis treated with *Allium sativum*. Cancer Ther..

[45] Estepa V., Ródenas S., Martín M.C. (2001). Optimización de un método para la determinación de la peroxidación lipídica en suero humano. Anal. Real Acad. Nac. Farm..

[46] Benzie I.F.F., Strain J.J. (1996). The ferric reducing ability of plasma (FRAP) as a measure of “antioxidant power”: The FRAP assay. Anal. Biochem..

